# EGFR Activation Impairs Antiviral Activity of Interferon Signaling in Brain Microvascular Endothelial Cells During Japanese Encephalitis Virus Infection

**DOI:** 10.3389/fmicb.2022.894356

**Published:** 2022-06-30

**Authors:** Ya-Ge Zhang, Hao-Wei Chen, Hong-Xin Zhang, Ke Wang, Jie Su, Yan-Ru Chen, Xiang-Ru Wang, Zhen-Fang Fu, Min Cui

**Affiliations:** ^1^State Key Laboratory of Agricultural Microbiology, College of Veterinary Medicine, Huazhong Agricultural University, Wuhan, China; ^2^Key Laboratory of Preventive Veterinary Medicine in Hubei Province, The Cooperative Innovation Center for Sustainable Pig Production, Wuhan, China; ^3^Key Laboratory of Development of Veterinary Diagnostic Products, Ministry of Agriculture of the People's Republic of China, Wuhan, China; ^4^International Research Center for Animal Disease, Ministry of Science and Technology of the People's Republic of China, Wuhan, China

**Keywords:** Japanese encephalitis virus, human brain microvascular endothelial cells, epidermal growth factor receptor, interferon, ERK

## Abstract

The establishment of Japanese encephalitis virus (JEV) infection in brain microvascular endothelial cells (BMECs) is thought to be a critical step to induce viral encephalitis with compromised blood–brain barrier (BBB), and the mechanisms involved in this process are not completely understood. In this study, we found that epidermal growth factor receptor (EGFR) is related to JEV escape from interferon-related host innate immunity based on a STRING analysis of JEV-infected primary human brain microvascular endothelial cells (hBMECs) and mouse brain. At the early phase of the infection processes, JEV induced the phosphorylation of EGFR. In JEV-infected hBMECs, a rapid internalization of EGFR that co-localizes with the endosomal marker EEA1 occurred. Using specific inhibitors to block EGFR, reduced production of viral particles was observed. Similar results were also found in an EGFR-KO hBMEC cell line. Even though the process of viral infection in attachment and entry was not noticeably influenced, the induction of IFNs in EGFR-KO hBMECs was significantly increased, which may account for the decreased viral production. Further investigation demonstrated that EGFR downstream cascade ERK, but not STAT3, was involved in the antiviral effect of IFNs, and a lowered viral yield was observed by utilizing the specific inhibitor of ERK. Taken together, the results revealed that JEV induces EGFR activation, leading to a suppression of interferon signaling and promotion of viral replication, which could provide a potential target for future therapies for the JEV infection.

## Introduction

Japanese encephalitis virus (JEV) is an arbovirus that remains the leading cause of Flavivirus-based mosquito-borne viral encephalitis worldwide. The epidemic of Japanese encephalitis (JE) is mainly observed in Asia, but the western Pacific and northern Australia are also exposed to the risk of JEV infection nowadays (Turtle and Driver, 2018; Kuwata et al., 2020). Approximately, one-third of patients hospitalized with JE die due to the lack of approved therapies and antiviral drugs in endemic areas, which is thought to be a high case fatality rate (Griffiths et al., 2014).

Japanese encephalitis is usually accompanied by the disruption of the blood–brain barrier (BBB), which is a highly specialized structure as the first immune barrier and helps in the maintenance of the homeostasis of the central nervous system (CNS) microenvironment by restricting the invasion of pathogens (Saunders et al., 2008; Burkhart et al., 2015; Chen and Li, 2021). Among the cells composing BBB, brain microvascular endothelial cells (BMECs) are the crucial components that space out neurons from blood circulation (Sweeney et al., 2019). The release of the viral particles from JEV-infected BMECs has been considered to be one of the mechanisms by which the JEV penetrates the BBB (Lai et al., 2012; Al-Obaidi et al., 2017). Apart from JEV, it has been reported that the Zika virus (ZIKV) can continuously establish infection and is released basolaterally from human brain microvascular endothelial cells (hBMECs) (Mladinich et al., 2017). The endothelial cells of the BBB expressed all of the recognized HCV entry receptors and supported HCV infection, which contributed to changed endothelial permeability (Fletcher et al., 2012). In terms of immune activation of BBB, previous studies provide little information on the antiviral ability of BMECs directly against JEV. Nevertheless, immune response featuring pro-inflammatory and anti-inflammatory signaling could be initiated in BMECs in response to pathogens or external stimuli (Chen and Li, 2021). A previous study showed that a robust interferon-β (IFN-β) response was induced in endothelial cells during Nipah virus (NiV) infection (Lo et al., 2010). Li's report also has revealed that the antiviral factors released from immune-activated hBMECs inhibit the infection of HIV in macrophages (Li et al., 2013). Besides, in hBMECs, productive JEV infection has been reported, which causes IFN-β and tumor necrosis factor-α (TNF-α) production (Shwetank et al., 2013). Without a doubt, exposure to pathogens like JEV could profoundly affect the immune function of BMECs.

The epidermal growth factor receptor is a tyrosine kinase receptor that belongs to the ERBB family, whose activation involves the specific ligands including epidermal growth factor (EGF), transforming growth factor-α (TGFα), betacellulin (BTC), heparin-binding EGF-like growth factor (HB-EGF), epiregulin (EREG), epigen (EPGN), and amphiregulin (AREG) (Cataldo et al., 2011; Liebmann, 2011; Freed et al., 2017). The activation of EGFR has been proved to be decisive in many diseases (Linggi and Carpenter, 2006; Finigan et al., 2012; Chen et al., 2017; Martin et al., 2017). Recent studies have demonstrated that activated EGFR in hBMECs contributes to bacteria-mediated disruption of BBB and causes neuroinflammation (Wang et al., 2016; Yang et al., 2016). The activation of EGFR and its downstream cascade are also essential in restricting the host's innate immunity against virus infection (Lupberger et al., 2011; Ueki et al., 2013). For example, impaired activation of EGFR by prostasin causes a decreased dengue virus (DENV) propagation through the downregulation of cyclooxygenase/prostaglandin-E2 (COX-2/PGE2) (Lin et al., 2019). EGFR signaling suppresses the production of IFN response genes to weaken the host antiviral effect in HCV infection (Lupberger et al., 2013). Similarly, Yang et al. have reported that infection by porcine epidemic diarrhea virus (PEDV) activates EGFR and its downstream STAT3, and the treatment with the inhibitor of either EGFR or STAT3 decreases virus production by the upregulation of type I interferon (IFN-I) (Yang et al., 2018). Moreover, during influenza A virus (IAV) infection, the EGFR/mitogen-activated protein kinase/extracellular signal-regulated kinase (MAPK/ERK)/specificity protein 1 (Sp1) signaling cascade was found to participate in the production of the epithelial cell-derived mucin MUC5AC, which provides a protective barrier against pathogenic challenges (Barbier et al., 2012). In the case of JEV, attachment of the virus to host cells induces the activation of EGFR signaling, thereby leading to RhoA activation, which promotes the activation of caveolin-1 and Rac1, thus resulting in caveolin-associated viral internalization (Xu et al., 2016). In hBMECs, activated EGFR has been reported not only to prompt the invasion of meningitic *Escherichia coli* (*E. coli*) but also to cause neuroinflammation in *Streptococcus suis* (*S. suis*) meningitis (Yang et al., 2016; Fu et al., 2018). However, to the best of our knowledge, whether EGFR is involved in the JEV infection of hBMECs is still obscure.

In the present study, we validated the specific role of EGFR on JEV propagation in brain microvascular endothelial cells. JEV infection activated EGFR and its downstream cascades. Using specific inhibitors and knocking out the endogenous EGFR, it was confirmed that EGFR assists the replication and virion production of JEV by negatively regulating the antiviral efficacy of interferon signaling, but does not affect viral attachment or entry. Together with the knowledge of the roles of EGFR in virus infection, the work presented here revealed the mechanism of how JEV exploits EGFR signaling to prompt virus replication, which is likely to provide insights into JEV-induced CNS invasion.

## Materials and Methods

### Viruses and Cell Culture

The JEV P3 strain was generated and harvested in mice brains according to the protocol in the previous study (Li et al., 2015). The heat-inactivated JEV P3 (heated-JEV P3) was acquired via incubating at 94°C for 15 min (Chang et al., 2015). The ZIKV MR766 was generated in the Vero cell line and collected for experiments. The hBMEC and hBMEC EGFR knockout (EGFR-KO) cell lines were kindly provided by Dr. Xiangru Wang (Huazhong Agricultural University, Wuhan, China) and subcultured in flasks with 10% heat-inactivated fetal bovine serum (FBS), 2 mM L-glutamine, 1 mM sodium pyruvate, vitamins, essential amino acids, nonessential amino acids, and 100 U/ml penicillin-streptomycin in RPMI 1640 medium, and maintained in a humidified incubator (37°C, 5% CO_2_) (Wang et al., 2016). The Vero and BHK-21 cells were cultured in Dulbecco's Modified Eagle's Medium (DMEM) (Gibco) plus 10% FBS and 1% penicillin/streptomycin. The cells were starved in a serum-free medium for 12~16 h and subjected to further experimentation when cells were 90~95% confluent.

### Reagents, Antibodies, and Inhibitors

Human EGF recombinant protein (rhEGF) was purchased from Life Technologies (Gaithersburg, MD USA). Proteinase K, phenylmethylsulfonyl fluoride (PMSF), and Cell Counting Kit (CCK-8) assay were all obtained from Biosharp (Anhui, China). HighGene Transfection reagent (RM09014) was purchased from ABclonal (Wuhan, Hube, China). The EGFR inhibitor AG1478 (HY-13524), Gefitinib inhibitor (HY-50895), ERK inhibitor U0126 (HY-12031), and HSP90/STAT3 inhibitor 17-AAG (HY-10211) were purchased from MedChem Express (Shanghai, China). Antibodies used for Western blotting, anti-phospho-EGFR (Tyr1068), and anti-EGFR antibodies (both rabbit polyclonal antibodies) were obtained from Cell Signaling Technology (Danvers, MA, USA). Anti-phospho-STAT3 (Tyr705) and anti-STAT3 (both rabbit) were purchased from Abcam (Cambridge, MA, USA). Anti-phospho-ERK-T202/Y204 and anti-ERK antibodies (both rabbit) were purchased from ABclonal (Wuhan, Hubei, China). An anti-β-actin antibody was purchased from Proteintech (Chicago, IL, USA). Monoclonal antibodies of JEV envelope (E) and ZIKV non-structural protein 5 (NS5) were courtesy of Dr. Shengbo Cao (Huazhong Agricultural University, Wuhan, China). For immunofluorescence, mouse anti-EGFR antibody was obtained from Abcam and rabbit anti-EEA1 antibody was from Cell Signaling Technology, while Alexa Fluor 488 or Cy3-labeled secondary antibodies and DAPI were obtained from Thermo Fisher Scientific (San José, CA, USA).

### Drug Treatments and Virus Infection

When cells reached 100% confluence, hBMECs and Vero cells were serum-starved for 12~16 h before rhEGF was added. After that, cells were washed to prepare for the subsequent experiments. For the inhibition experiment, hBMECs were treated with the inhibitors AG1478, gefitinib, U0126, 17-AAG, or the vehicle control dimethyl sulfoxide (DMSO) at various concentrations for the corresponding times as previously described (András et al., 2005; Maruvada and Kim, 2012). For the virus infection procedure, hBMECs and Vero cells were infected with the JEV at a multiplicity of infection (MOI) of 1 at various times. All virus infection experiments were executed using the above-described procedure unless otherwise stated. The treated cells were then acquired for the following analysis.

### RNA Extraction and Quantitative Real-Time PCR

Total RNA from treated cells was extracted with TRIzol reagent following the manufacturer's instructions (Invitrogen, Grand Island, NY). The RNA was reverse-transcribed into cDNA using the ReverTra Ace qPCR RT kit (Toyobo, Japan). SYBR Green 2 × mix (Invitrogen) was utilized to perform quantitative real-time PCR using a 7500 Real-Time PCR System (Applied Biosystems). The transcriptional levels of the target mRNA were normalized to β-actin except for the JEV-C gene and ZIKV-NS5 gene. A standard curve was generated to quantify the viral copy numbers, and the pcDNA3.0-HA/JEV-C gene plasmid or the pcDNA3.0-HA/ZIKV-NS5 gene plasmid was used as a template. All amplifications were performed in triplicate, and primers employed for the quantitative real-time PCR are listed in Table 1.

**Table 1:** Primers used for qPCR in this study.

**Gene**	**Forward (5**^′^**- 3**^′^**)**	**Reverse (5**^′^**- 3**^′^**)**	**Species**
β-actin	AGCGGGAAATCGTGCGTGAC	GGAAGGAAGGCTGGAAGAGTG	Human
EGFR	TACAGACCCAAGAGCAGCA	AGCCGTACATAGATCCAGAA	Human
IFN-α	FCTTGTGCCTGGGAGGTTGTC	TAGCAGGGGTGAGAGTCTTTG	Human
IFN-β	GACGCCGCATTGACCATCTA	TTGGCCTTCAGGTAATGCAGAA	Human
IFN-λ1	CACATTGGCAGGTTCAAATCTCT	CCAGCGGACTCCTTTTTGG	Human
IFN-λ2,3	CTGACGCTGAAGGTTCTGGAG	CGGAAGAGGTTGAAGGTGACAG	Human
ISG15	CGCAGATCACCCAGAAGATCG	TTCGTCGCATTTGTCCACCA	Human
JEV-C	GGCTCTTATCACGTTCTTCAAGTTT	TGCTTTCCATCGGCCYAAAA	
ZIKV-NS5	TGCTCTCAACACATTCACCAACTTG	CATCTCCACTGACCGCCATTCG	

### Immunofluorescence (IF) and Western Blotting (WB)

Immunofluorescence staining of the cells cultured in the 12-well plate was performed to determine virus replication. In brief, the cells were rinsed with 1 × PBS and fixed in 4% paraformaldehyde. The fixed cells were subsequently permeabilized and blocked with 0.2% Triton X-100 and 5% bovine serum albumin (BSA) in 1 × PBS for 2 h at room temperature, followed by incubation with mouse anti-JEV-E or mouse anti-ZIKV-NS5 monoclonal antibody (1:1,000) in a humidified chamber overnight at 4°C. After three washes with 1 × PBS, the cells were incubated with fluorescently labeled anti-mouse IgG for 1 h at room temperature. Then, the cell nuclei were stained with DAPI. Finally, immunostained samples were visualized under fluorescence microscopy or laser confocal microscopy (Nikon STORM).

For Western blotting analyses, briefly, cells were washed with ice-cold 1 × PBS and then whole cell extracts were prepared using 60 μl of RIPA buffer containing protease inhibitor cocktail (Roche) and phosphatase inhibitor cocktail (Roche). Then the lysates were centrifuged and quantified for protein concentration using a BCA protein assay kit (Beyotime, China). Protein samples were separated by sodium dodecyl sulfate-polyacrylamide gel electrophoresis (12%) and then transferred to PVDF membranes using a wet transfer system. The membranes were blocked in 5% BSA in Tris-buffered saline with Tween 20 (TBST) for 1 h at room temperature and were incubated with primary antibodies overnight at 4°C with shaking. After washing thrice with TBST, the membrane was incubated with a horseradish peroxidase (HRP)-labeled secondary antibody and finally visualized.

### Plaque Assay and CCK-8 Assay

Plaque assays were performed on BHK-21 cells utilizing the 24-well culture plate. The supernatants were collected from virus-infected cells at indicated time intervals and stored at −80°C after removing cellular debris. The clarified supernatant was subjected to serial dilution and added into confluent BHK-21 cells for 1.5 h, and then the cells were overlaid with DMEM containing 1.5% carboxymethylcellulose (Sigma-Aldrich) for 5 days. Finally, the plaques were counted to calculate viral titers.

The CCK-8 assay was performed to determine cell viability. Briefly, the hBMECs were plated into a 96-well plate at a density of 5,000 cells per well for 24 h and starved for 12~16 h with a serum-free medium. The inhibitor of various concentrations was added into cells for 2 h, and CCK-8 solutions were subsequently added to each well and continuously incubated for approximately 45 min. Finally, the absorbance of the samples was measured at a wavelength of 450 nm in a Spectrophotometer reader (Bio-Rad, CA, USA).

### Measurement of Virus Attachment and Entry

The hBMECs were seeded in the 24-well plate until reaching 100% confluence, and the incubation medium was replaced with serum-free DMEM for 12~16 h. Then, the cells were incubated with JEV of indicated MOI at 4°C for 1 h (attachment assay). For entry assay, the plate was shifted to 37°C containing 5% CO_2_ for 2 h, proteinase K (1 mg/ml) was used to remove non-internalized virions, and then 2 mM PMSF in 1 × PBS with 3% bovine serum albumin was applied to inactivated proteinase K as previously described (Dejarnac et al., 2018).

### Statistical Analysis

The statistical data are presented as the mean ± SEMs values, with at least three replicates for each treatment. Student's *t*-test, one-way analysis of variance (ANOVA), or two-way ANOVA were applied to analyze the statistical significance of the differences by using GraphPad Prism (v7.0; GraphPad, La Jolla, CA, USA). A value of *p* < 0.05 (^*^) was considered statistically significant, while values of *p* < 0.01 (^**^), *p* < 0.001 (^***^), and *p* < 0.0001 (^****^) indicated extremely significant differences.

## Results

### JEV Induced EGFR Activation at the Early Phase of Infection

**Figure 1:**
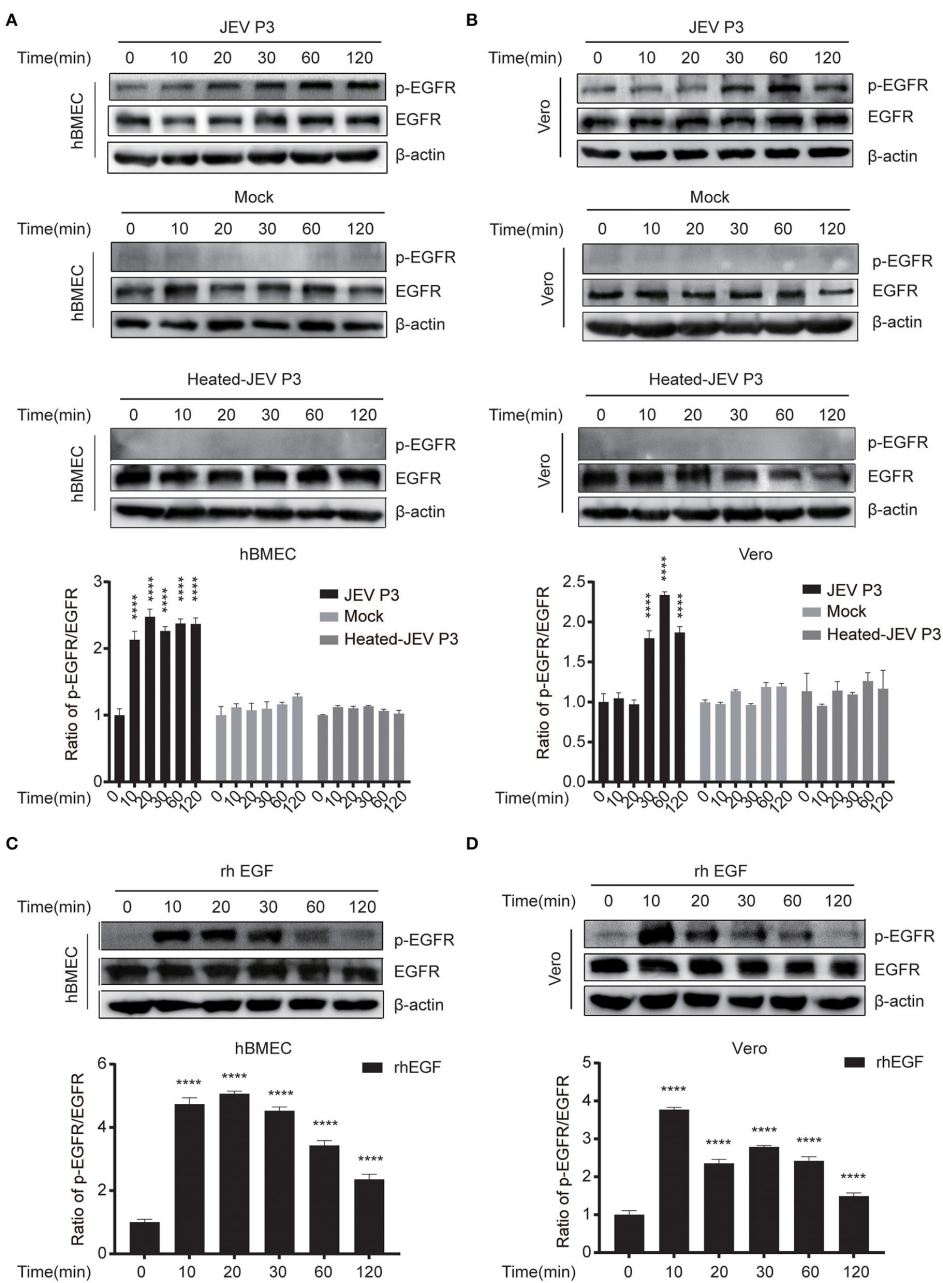
JEV infection induces EGFR phosphorylation. **(A,B)** hBMECs and Vero cells were infected with JEV P3 (MOI = 1) or heated-inactivated JEV P3 (heated-JEV P3, 94°C, 15 min, MOI = 1) for 0, 10, 20, 30, 60, and 120 min, and the cells treated with DMEM were used as control. Cell lysates were harvested and subsequently examined by Western blotting using the antibodies of p-EGFR, EGFR, and β-actin. Quantification of phosphorylated levels of EGFR relative to the total EGFR levels was performed. **(C,D)** rhEGF-treated cells showed increased phosphorylation of EGFR in a time-dependent manner. About 10 ng/ml rhEGF was added into hBMECs and Vero cells for indicated times in a 37°C humidified incubator after serum-starved for 12~16 h, then the cells were collected and prepared for Western blotting. Quantification of phosphorylated levels of EGFR relative to the total levels was analyzed with ImageJ and presented as percent of the control samples. The results are represented as mean ± SEM values from three independent experiments. ****, *p* < 0.0001.

According to the IFN-related genes identified to be markedly altered in succession at three time points in our unpublished hBMECs RNA-seq data and similar results in Li's report on RNA-seq of JEV-infected mouse brain (Supplementary Figure S1A) (Li et al., 2017), a protein to protein interaction (PPI) network was constructed by STRING, from which the emergence of EGFR caught our attention (Supplementary Figure S1B). Nevertheless, in the monolayer of hBMECs, there was no significant difference both in transcription and translation levels of EGFR at 12, 36, and 72 h post-infection (hpi) (Supplementary Figure S1C). Many viruses activate EGFR through phosphorylation at the early stage of infection, including ZIKV, PEDV, and IAV (Yang et al., 2018; Sabino et al., 2021; Wang et al., 2021). To figure out whether EGFR could be activated by JEV through phosphorylation, phosphorylation on tyrosine sites of EGFR in hBMECs at the early phase of JEV infection was examined. hBMECs were treated with JEV, heat-inactivated JEV (heated-JEV), and DMEM, respectively, and phosphorylated EGFR was determined by Western blotting at 0, 10, 20, 30, 60, and 120 min post-treatment. Simultaneously, the total EGFR expression levels were also measured. The accumulation of phosphorylated EGFR appeared at 10 min and was sustained for at least 2 h in JEV-infected hBMECs but not in heated-JEV or mock-infected hBMECs (Figure 1A). The inactivation of heated-JEV was verified by plaque assay, in which no live virus particles were detected (data not shown). Next, Vero cells were utilized to determine whether the activation of EGFR occurred in other cell types besides hBMECs. It was observed that the phosphorylation level of EGFR was also boosted in Vero cells, which is similar to the result in hBMECs (Figure 1B). These results demonstrated that JEV but not heated-JEV could induce the phosphorylation of EGFR with no remarkable effect on the expression of total EGFR. EGF, one of the ligands of EGFR, was utilized as a positive stimulator in the activation of EGFR (Yang et al., 2018; Kim et al., 2020). To determine if cells are responsive to EGF, cells were treated with rhEGF, which could prompt the receptor dimerization, autophosphorylation, and activation of EGFR (Herbst, 2004; Xiong et al., 2020). The phosphorylation of EGFR was induced at 10 min and reached the peak at 30 min in hBMECs, but the highest level was around 10 min in Vero cells, and the total EGFR showed no noticeable change over time (Figures 1C,D). The divergence of the highest levels of phosphorylated EGFR in hBMECs and Vero cells is probably owing to the different biological characteristics of the two cell types, while both cells are consistent with the previous report that prolonged stimulation with EGF leads to the degradation of ligand-induced phosphorylated EGFR (Schlessinger, 1986; Sorkin, 2001). Taking all these observations together, these results suggested that JEV infection activates EGFR through phosphorylation at the early stage of infection.

### JEV Triggers EGFR Internalization in hBMECs

**Figure 2:**
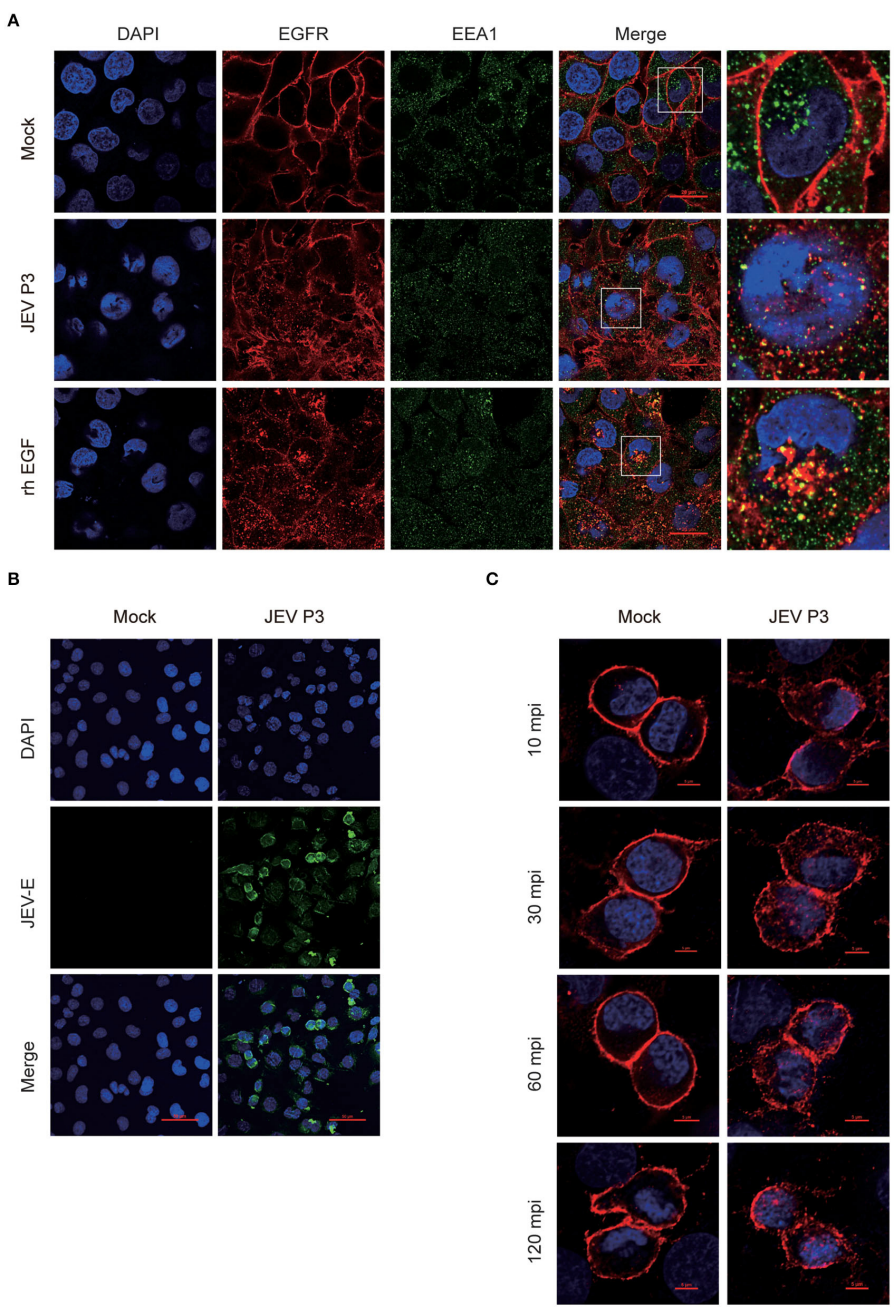
Internalization of EGFR in JEV-infected hBMECs. hBMECs were infected with JEV P3 (MOI = 1) for 16 h, and then images were captured by confocal laser scanning microscopy. **(A)** Uninfected cells and rhEGF-stimulated cells (10 ng/ml rhEGF for 30 min) were utilized as negative and positive controls, respectively. Cells were fixed and stained with specific antibodies (EGFR and EEA1), and nuclei were stained with DAPI. Bar, 20 μm. **(B)** Negative control was presented with uninfected cells. Nuclei were stained with DAPI, and JEV-E protein was visualized with a JEV envelope protein monoclonal antibody. Bar, 50 μm. **(C)** hBMECs were exposed to JEV P3 (MOI = 10) or mock-treated for 60 min at 4°C and then transferred to a 37°C incubator for 10, 30, 60, and 120 min. Uninfected cells were used as a negative control. Nuclei were stained with DAPI, and EGFR was visualized with a specific antibody. Bar, 5 μm. The results are represented as mean ± SEM values from three independent experiments.

To explore whether the intracellular localization of EGFR could be affected by JEV, hBMECs were infected with JEV at an MOI of 1. Simultaneously, hBMECs were treated with rhEGF as a positive control. The specific antibodies of EGFR (red) and EEA1 (endosome marker, green) were utilized to determine the localization of EGFR under JEV infection, which was observed by confocal laser scanning microscopy. As shown in Figure 2A, compared to hBMECs in the mock group, both in JEV- and rhEGF-treated hBMECs, cell membrane EGFR substantially decreased and EGFR in cell cytoplasm and nucleus increased and presented as intracellular dot-like structures, which is concomitant with the increasing co-localization of EGFR and endosomal marker EEA1 (Figure 2A). Thereafter, the infection of JEV in hBMECs was measured with JEV E-specific antibody (green) by confocal laser scanning microscopy (Figure 2B). These results suggested that EGFR is internalized after JEV infection. Previous data demonstrated that EGFR localization might be changed at the early stage of JEV infection. To further confirm the result, hBMECs were infected with JEV at an MOI of 10, and then images were captured by confocal microscopy. The majority of the EGFR is located at the plasma membrane in uninfected hBMECs, while contrastingly, the dot-like structures near the cytoplasm and nucleus appeared at 30 min and persisted up to 120 min after JEV infection (Figure 2C). The altered subcellular localization of EGFR in hBMECs indicates that EGFR is internalized at the early phase of JEV infection.

### The Inhibition of EGFR Phosphorylation Restricts the Production of Viral Particles in hBMECs

**Figure 3:**
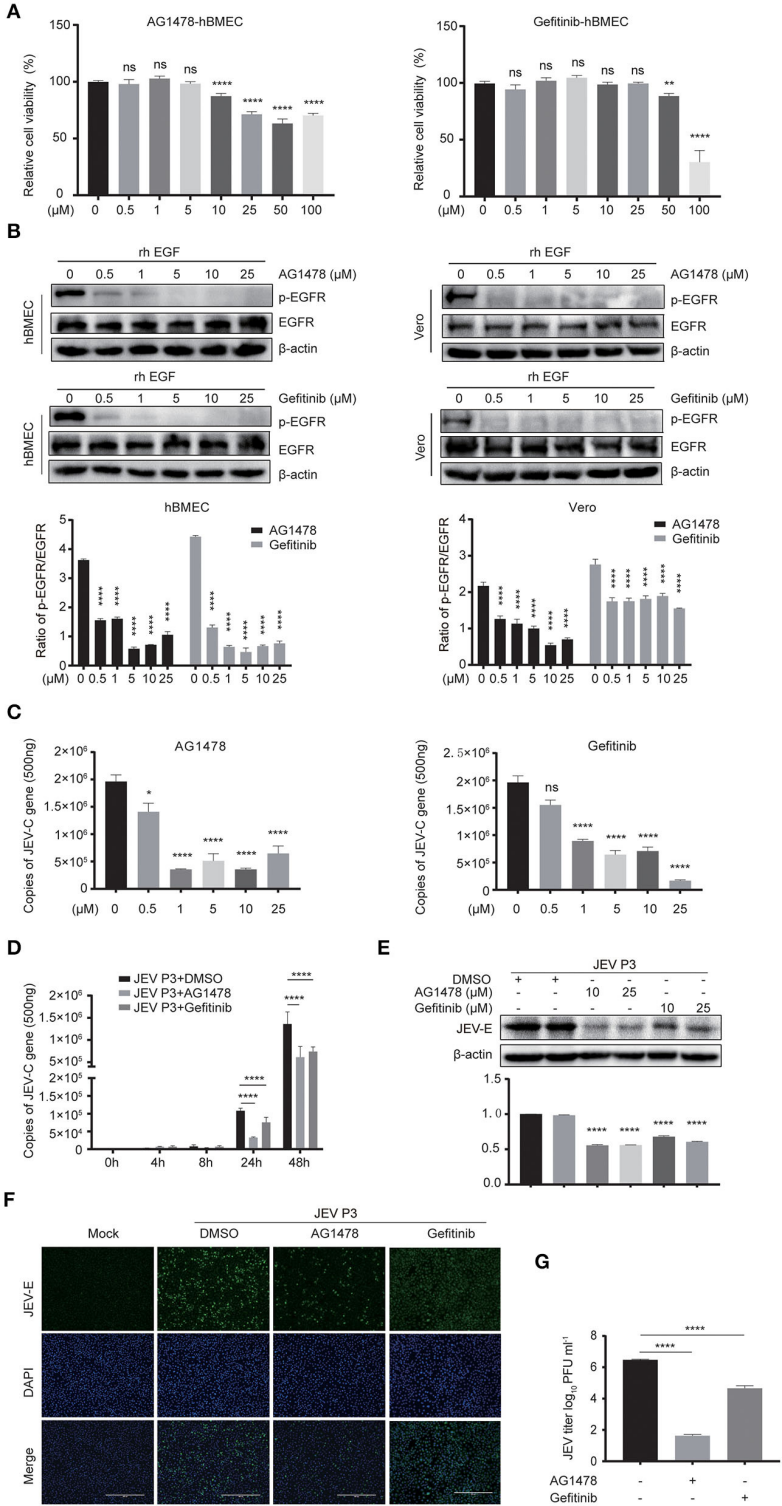
EGFR inhibitors reduce JEV infection in hBMECs. **(A)** Cell cytotoxicity of the EGFR inhibitors. hBMECs were treated with the carrier control DMSO, AG1478, or gefitinib at various concentrations for 48 h, and cell cytotoxicity was analyzed with the CCK-8 assay. **(B)** The inhibitory effect of AG1478 and gefitinib in rhEGF-treated cells. The hBMECs and Vero cells were treated with corresponding concentrations of AG1478 and gefitinib for 2 h and then rhEGF was added for another 30 min for hBMECs and 10 min for Vero cells, and subsequently, the lysed cells were subjected to Western blotting. hBMECs were pretreated with DMSO, AG1478, or gefitinib for 2 h followed by virus (MOI = 1) infection for corresponding times. Results were quantified. **(C,E)** The verification of the JEV P3 replication in RNA and protein levels treated with indicated concentrations of AG1478 and gefitinib for 24 h. Results were quantified. **(D)** The effects of 10 μM AG1478 or 25 μM gefitinib on JEV P3 replication in hBMECs over time with quantitative real-time PCR. **(F)** The immunofluorescence (IF) assay was performed to determine JEV P3 infection in hBMECs at 24 h after being treated with 10 μM AG1478 or 25 μM gefitinib. Cells were fixed and stained with a specific antibody (JEV-E protein monoclonal antibody), and nuclei were stained with DAPI. Scale bar, 400 μm. **(G)** Supernatants in JEV P3-infected hBMECs with different treatments were collected, and the viral titer was detected in BHK-21 cells with plaque assay. The results are represented as mean ± SEM values from three independent experiments. * *p* < 0.05; ** *p* < 0.01; ****, *p* < 0.0001; ns, not significant.

For further study, two EGFR tyrosine kinase inhibitors, AG1478 and gefitinib, were applied, which were functionally approved in the previous research (Dhar et al., 2018; Li et al., 2021). To determine the toxic effects of the inhibitors, the cell viability of hBMECs was assessed upon inhibitor treatment. The hBMECs were seeded in 96-well plates and treated with AG1478 or gefitinib with concentrations from 0.5 to 100 μM for 48 h. In gefitinib-treated hBMECs, there were still more than 70% of cells that remained viable at the dose of 50 μM; for AG1478 treatment, in the concentration of 50 μM, 90% of cells remained viable. Hence, based on the CC_50_ (cell cytotoxicity at 50%), 25 μM was chosen as the highest dose of treatment in the following study for both inhibitors (Figure 3A). Afterward, hBMECs and Vero cells were pretreated with AG1478 or gefitinib in different concentrations. It was observed that the tyrosine phosphorylation of EGFR abrogated markedly after pretreatment, which confirmed that AG1478 and gefitinib could be used as inhibitors of EGFR phosphorylation in both hBMECs and Vero cells (Figure 3B).

Previous studies gave evidence that the EGFR pathway plays a role in producing various viruses, such as DENV, ZIKV, etc (Ueki et al., 2013; Lin et al., 2019; Chuang et al., 2020). To explore whether EGFR is involved in the JEV infection process, cells were pretreated with AG1478 or gefitinib in different concentrations. It was observed that the level of viral RNA (JEV-C gene) was dramatically decreased in hBMECs treated with 0.5 μM of AG1478, 1 μM of gefitinib, or higher doses (Figure 3C). Further study demonstrated that both 10 μM AG1478 and 25 μM gefitinib memorably inhibited viral replication at 24 h and 48 h after JEV infection (Figure 3D). Consistent with the transcription levels, viral protein (JEV-E) expression was downregulated in cells treated with 10 μM AG1478 or 25 μM gefitinib at 24 hpi (Figure 3E). As shown in the immunofluorescence assay, the number of JEV-positive cells was significantly less in AG1478- or gefitinib-treated hBMECs than in a mock-treated control group (Figure 3F). Furthermore, the plaque assay illustrated that the production of viral particles was markedly reduced with inhibitor treatment in hBMECs (Figure 3G). Then, we investigated whether activated EGFR could also affect the infection of other CNS-invading flaviviruses in hBMECs. It was reported that ZIKV persistently infects hBMECs, and another report revealed that EGFR is related to the ZIKV life cycle (Mladinich et al., 2017; Sabino et al., 2021). Thus, the replication of ZIKV was determined in AG1478- and gefitinib-treated hBMECs. The result showed a significant reduction in the expression of ZIKV protein (NS5) in the inhibitor-treated groups compared to that in the mock group, which is consistent with the result of JEV infection (Supplementary Figures S2A,B). Taken together, the results demonstrated that activated EGFR is associated with the infection of JEV and ZIKV in hBMECs.

### Viral Infection Is Impaired in Endogenous EGFR Knockout hBMECs

**Figure 4:**
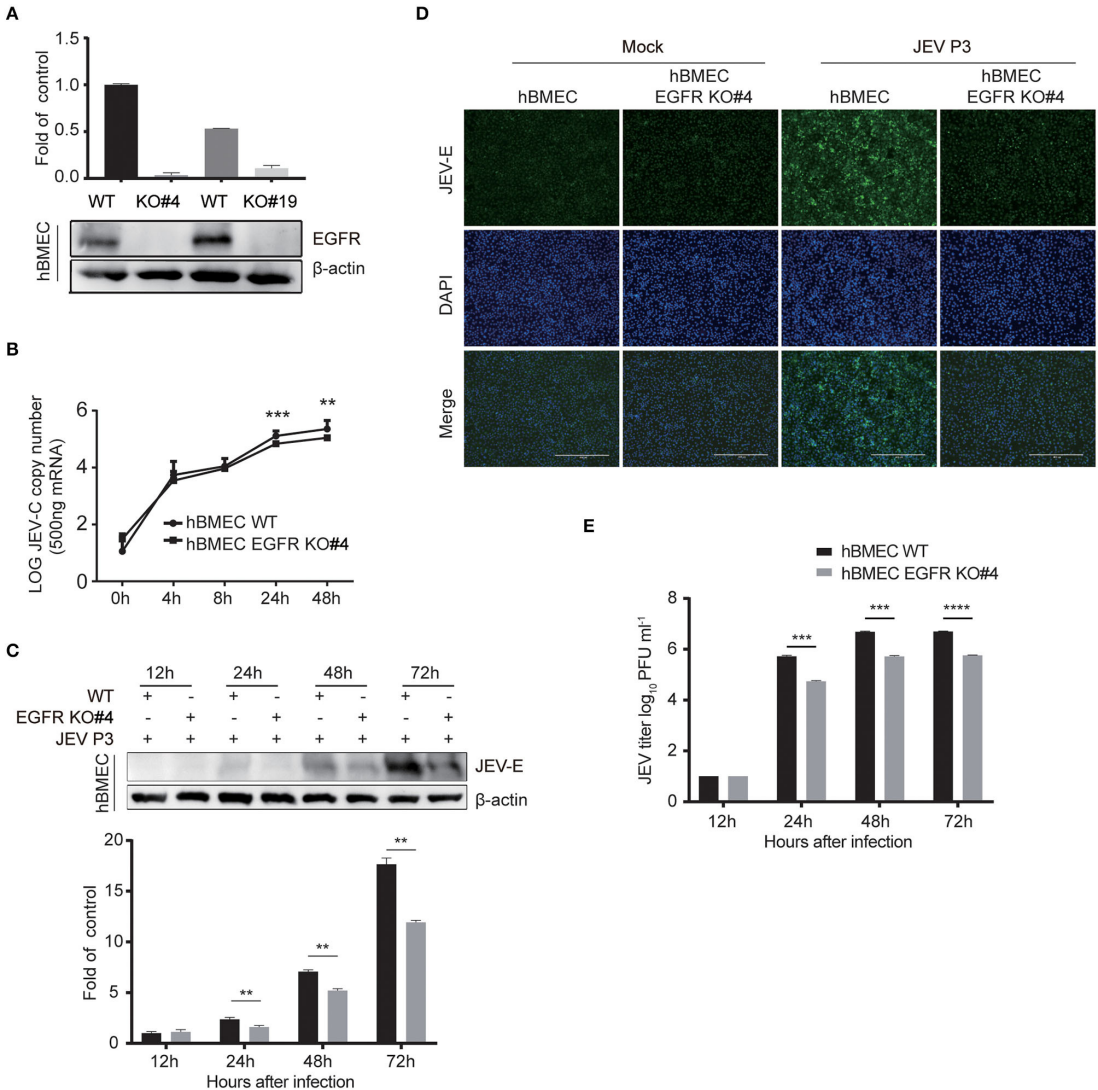
Viral infection was reduced in EGFR knockout hBMECs. **(A)** The verification of the EGFR knockout was measured with Western blotting in hBMECs. Results were quantified. **(B)** The comparison of JEV P3 replication between wild-type and EGFR-KO hBMECs over time by quantitative real-time PCR. **(C)** Western blotting assay was performed to determine the expression of JEV-E protein at different times in wild-type and EGFR-KO hBMECs. Results were quantified. **(D)** Wild-type and EGFR-KO hBMECs were infected with JEV P3 for 24 h, and then immunofluorescence (IF) staining was performed to detect JEV-E protein with JEV-E protein monoclonal antibody. Nuclei were stained with DAPI. Scale bar, 400 μm. **(E)** Supernatants in JEV P3-infected wild-type and EGFR-KO hBMECs were collected over time, and the viral titer was detected in BHK-21 cells by plaque assay. The results are represented as mean ± SEM values from three independent experiments. **, *p* < 0.01; ***, *p* < 0.001; ****, *p* < 0.0001.

The above-mentioned results showed a positive correlation between the activated EGFR and viral infection in hBMECs. To further evaluate the function of EGFR in JEV infection, EGFR knockout (KO) hBMEC cell lines were utilized, which were previously generated by using clustered regularly interspaced short palindromic repeat (CRISPR)/Cas9-mediated gene editing method (Fu et al., 2018). First, the expression of EGFR was confirmed in EGFR-KO cells by Western blotting (Figure 4A). The viral infection was drastically reduced in EGFR-KO cells both in RNA and protein levels in a time-dependent manner compared to that observed in wild-type (WT) hBMECs (Figures 4B,C). As shown in the immunofluorescence assay, the number of JEV-positive cells was significantly decreased in EGFR-KO hBMECs than in WT hBMECs (Figure 4D). Additionally, the plaque assay showed the viral titer was much lower in the supernatant of EGFR-KO hBMECs than that of WT hBMECs at 24, 48, and 72 hpi, which is consistent with the result of RNA and protein levels (Figure 4E). Furthermore, similar to JEV infection, the depression of ZIKV infection was also found in EGFR-KO hBMECs (Supplementary Figures S3A–C). These findings support the proposition that EGFR is crucial in mediating Flaviviridae infection in hBMECs.

### EGFR Is Related to the Antiviral Response of Interferon Signaling

**Figure 5:**
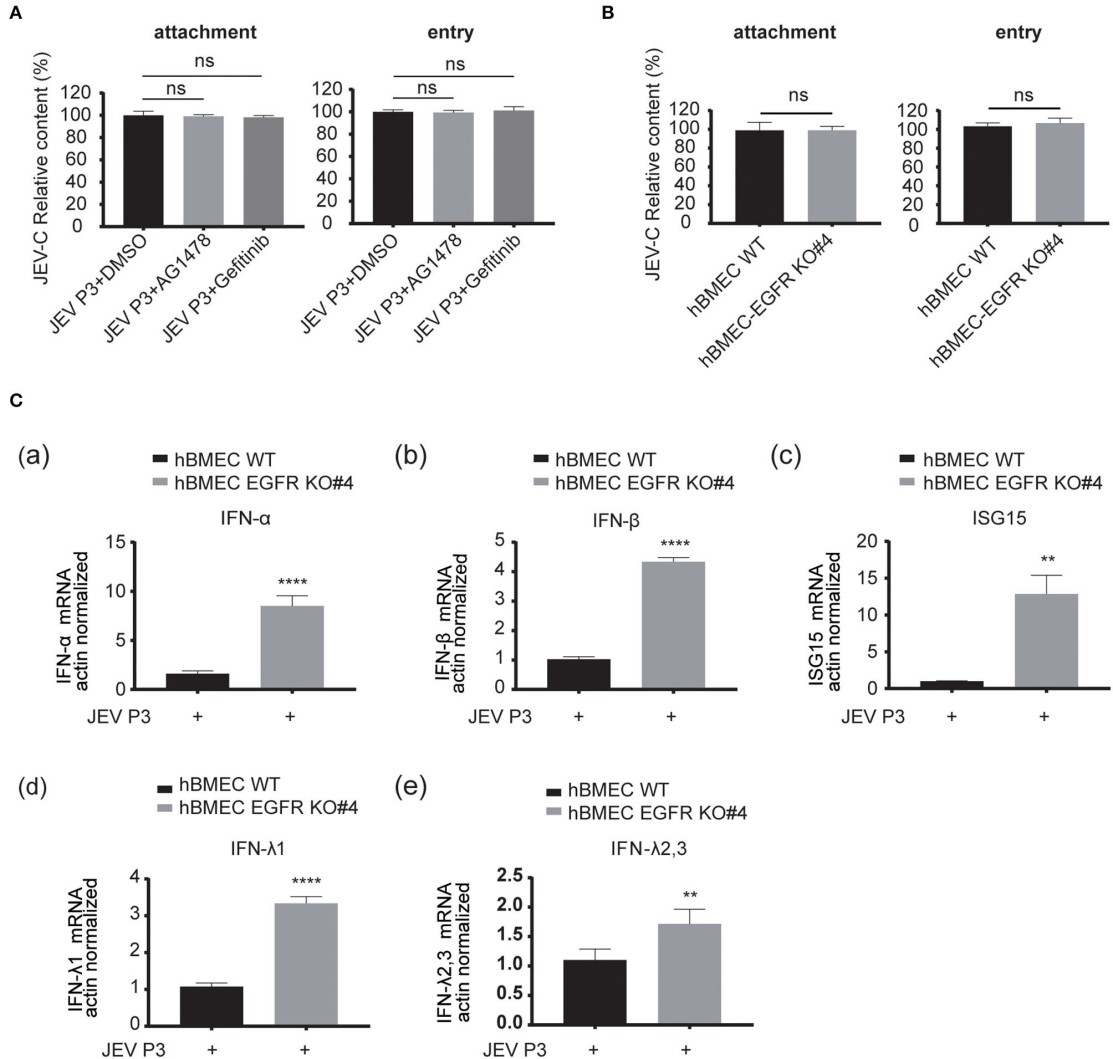
EGFR is involved in the antiviral response of interferon signaling. **(A)** The hBMECs were treated with AG1478, gefitinib, or vehicle control DMSO, and then bound with JEV P3 (MOI = 10) at 4°C for 1 h for attachment. The cells were transferred to a 37°C incubator for 1 h for entry, and total RNA was extracted to determine the RNA copy numbers of JEV P3 with quantitative real-time PCR. **(B)** The hBMECs and EGFR-KO hBMECs were infected with JEV P3 (MOI = 10) at 4°C for 1 h. After washing thrice with PBS containing proteinase k, the cells were then transferred to a 37°C incubator for 1 h to allow virus entry. **(C)** Comparison between hBMECs and EGFR-KO hBMECs in mRNA levels of IFN (IFN-α, IFN-β, IFN-λ1, and IFN-λ2, 3) and ISG15 production in the presence or absence of JEV P3 infection. The hBMECs and EGFR-KO hBMECs were infected with JEV P3 (MOI = 1) for 8 h, and total RNA was extracted and quantitative real-time PCR was performed. The results are represented as mean ± SEM values from three independent experiments. **, *p* < 0.01; ****, *p* < 0.0001; ns, not significant.

As EGFR is positively correlated with viral infection in hBMECs, the underlying specific mechanism needs to be further elucidated. The previous report had identified EGFR as a critical regulator in JEV entry into human neuronal cells (Xu et al., 2016). Here, RT-qPCR was performed to validate whether EGFR is related to JEV entry into hBMECs. However, pretreatment with EGFR inhibitor AG1478 or gefitinib did not change either the attachment or entry of JEV to hBMECs compared to the control group treated with DMSO (Figure 5A). Additionally, consistent with inhibitor treatment, no significant difference was observed in viral attachment and entry between EGFR-KO hBMECs and WT hBMECs (Figure 5B). Since EGFR does not affect JEV attachment and entry in hBMECs, how does EGFR facilitate JEV infection in hBMECs? It has been widely reported that EGFR participates in the regulation of host innate immunity during viral infection (Lupberger et al., 2013; Qiu et al., 2020; Wang et al., 2021). For example, respiratory virus (RSV) induced EGFR phosphorylation, which inhibited interferon regulatory factor 1 (IRF1)-regulate interferon-lambda (IFN-λ) production and breakdown of airway epithelium antiviral response (Kalinowski et al., 2018). Besides, in COVID-19 therapy, EGFR signaling inhibitors potentiated the IFN-I response, thereby considered to be an attractive therapeutic strategy (Matsuyama et al., 2020). On the other hand, regarding the tight correlation between interferon response and JEV infection, it was also reported that interferon signaling affects the inoculation dose-independent mortality in JEV attacked mice (Aoki et al., 2014). Besides, restricted viral propagation was observed when IFN-β production was enabled by neutralizing miR-301a in mouse neurons (Hazra and Kumawat, 2017), *In vitro*, porcine IFN-α inhibited JEV replication, and the overexpression of ISG15 showed antiviral activity against JEV infection (Hsiao et al., 2010; Liu et al., 2013). Based on the studies and previous PPI analysis (Supplementary Figure S1B), we speculated that EGFR might serve as a regulator in interferon signaling of host innate immune response in JEV-infected hBMECs. Hence, the expression of several interferons and interferon-stimulated genes (ISGs) was measured. The result showed that the gene expression of type I IFNs (IFN-α and IFN-β), type III IFNs (IFN-λ1, and IFN-λ2, 3), and ISG15 were significantly increased in EGFR-KO hBMECs compared to the WT hBMECs under JEV infection (Figure 5C). Collectively, these data demonstrated the negative correlation between EGFR and interferon signaling in JEV-infected hBMECs.

### ERK Is Downstream of EGFR in Regulating Interferon Signaling During JEV Infection

**Figure 6:**
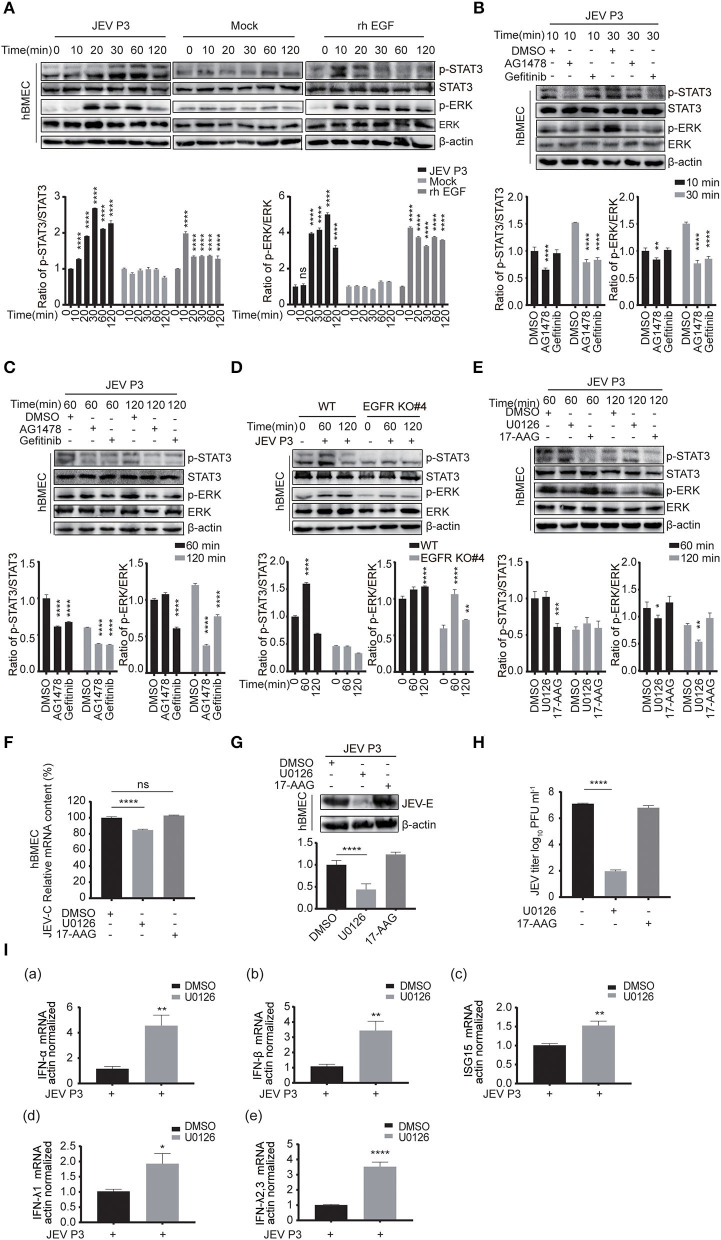
ERK signaling participated in EGFR-regulated host antiviral response by activating interferon signaling. **(A)** Both JEV P3 and rhEGF induced the phosphorylation of ERK and STAT3 in hBMECs. hBMECs were treated with JEV P3, rhEGF, or DMEM for 0, 10, 20, 30, 60, and 120 min. The expression of p-ERK, ERK, p-STAT3, and STAT3 were determined by Western blotting analysis with specific antibodies, while the expression of β-actin was detected as a loading control. Results were quantified. **(B,C)** hBMECs were pretreated with 10 μM AG1478, 25 μM gefitinib, or DMSO for 2 h, followed by JEV P3 infection (MOI = 1) for corresponding times, and the cell lysis was harvested. Western blotting assay was performed to detect the expression of p-EGFR, EGFR, p-STAT3, STAT3, p-ERK, and ERK. Results were quantified. **(D)** The hBMECs and EGFR-KO hBMECs were infected with JEV P3 (MOI = 1) for corresponding times, and the protein expression was examined using indicated antibodies. Results were quantified. **(E)** hBMECs were pretreated with U0126 (10 μM, ERK inhibitor), 17-AAG (1 μM, STAT3 inhibitor), or DMSO, and then infected with JEV P3 (MOI = 1) for 60 or 120 min. The proteins were analyzed with indicated antibodies by Western blotting. (F-H) hBMECs were pretreated with U0126 (10 μM), 17-AAG (1 μM), or DMSO followed by JEV P3 (MOI = 1) infection for 24 h, and the viral RNA levels **(F)**, the protein levels **(G)**, and the production of viral particles **(H)** were detected with quantitative real-time PCR, Western blotting, and plaque assay, respectively. Results were quantified. **(I)** hBMECs were pretreated with 10 μM U0126, followed by JEV P3 (MOI = 1) infection for 8 h, and then total RNA was collected and subjected to quantitative real-time PCR for assessing the expression levels of IFN (IFN-α, IFN-β, IFN-λ1, and IFN-λ2, 3) and ISG15. The results are shown as mean ± SEM values. Data are representative of three independent experiments. *, *p* < 0.05; **, *p* < 0.01; ***, *p* < 0.001; ****, *p* < 0.0001, ns, not significant.

Next, the downstream signaling of EGFR stimulated by JEV was investigated. As previously reported, the phosphorylation of ERK or signal transducers and activators of transcription 3 (STAT3) signaling was downstream of EGFR cascade to virus infection (Kung et al., 2011; Xu et al., 2013; Ding et al., 2017). Therefore, the phosphorylation of STAT3 and ERK was measured by Western blotting in hBMECs treated with JEV, rhEGF, or DMEM. Phosphorylation levels of STAT3 and ERK were increased in the hBMECs treated with JEV and rhEGF but not DMEM (Figure 6A). The decreased phosphorylation of STAT3 and ERK was observed at 30 min post-infection with the treatment of the inhibitor AG1478 and gefitinib, but not at 10 min (Figure 6B). It was assumed that the effects of EGFR on STAT3 and ERK may be induced later. Thus, the phosphorylation of STAT3 and ERK in hBMECs was determined at 60 and 120 min with the treatment of AG1478 or gefitinib. Decreased phosphorylation of STAT3 and ERK appeared in EGFR inhibitor-treated hBMECs and EGFR-KO hBMECs compared to the WT hBMECs (Figures 6C,D).

The use of 17-AAG, an inhibitor of heat shock protein 90 (HSP90), or U0126, an ERK inhibitor, has been shown to disrupt the phosphorylation of STAT3 or ERK, respectively, in hBMECs (Maruvada and Kim, 2012; Zhao et al., 2017). To further verify that STAT3 and ERK are related to virus replication during JEV infection, these two inhibitors were applied to the cells to inhibit phosphorylation of STAT3 or ERK (Figure 6E). When compared to mock-treated cells, a markedly impaired production of JEV viral RNA and E protein, as well as viral particles in U0126, was observed, but this change was not noticed in 17-AAG-treated hBMECs (Figures 6F–H). Previous reports have demonstrated that ERK negatively regulates the interferon signaling-mediated antiviral response (Yang and Ding, 2019; Freed et al., 2021). Therefore, the expression of interferons and ISGs was also measured with U0126 treatment in JEV-infected hBMECs. The expression levels of genes ISG15, IFN-α, IFN-β, IFN-λ1 and IFN-λ2, 3 were significantly increased in U0126-treated hBMECs compared to control during JEV infection, which probably is responsible for the dropped replication of JEV in hBMECs (Figure 6I). These findings support that the involvement of the EGFR-ERK pathway represses the host interferon signaling, leading to an acceleration of viral replication in hBMECs.

## Discussion

As the physical barrier between CNS and circulatory system, BMECs are the first line of BBB controlling the trafficking of JEV and/or JEV-infected cells into the CNS. The infection of BMECs by JEV is thought to be a pivotal step in causing the leakage of BBB, leading to brain infection and encephalitis (Lai et al., 2012; Al-Obaidi et al., 2017). Though BMECs have been largely known to compose BBB, their distinct antimicrobial and antiviral properties are also essential. In the present work, for the first time, the activation of the EGFR/ERK pathway was found, which negatively regulates the antiviral response of interferon signaling, promoting JEV replication in hBMECs.

As a member of the tyrosine kinase family, the biological significance of EGFR has been well studied in tumorigenesis. In recent years, the activated EGFR also appeared in response to viral infection, including IAV, PEDV, ZIKV, and severe acute respiratory syndrome coronavirus 2 (SARS-CoV-2) (Yang et al., 2018; Klann et al., 2020; Sabino et al., 2021; Wang et al., 2021). The activation of EGFR might build a favorable cellular environment to prompt viral entry or replication (Oshiumi et al., 2015; Fukano et al., 2021). In the case of JEV infection, the activated EGFR-PI3K signaling cascade was reported to play a crucial role in caveolin-1-mediated JEV entry into human neuronal cells (Xu et al., 2016). In the present study, the activated EGFR and its downstream signaling also appeared in the early phase of JEV-infected hBMECs. EGFR internalization has been identified in ZIKV-, HBV-, IAV-, and TGEV-infected cells, which assisted in efficient viral entry (Eierhoff et al., 2010; Hu et al., 2018; Iwamoto et al., 2019; Sabino et al., 2021). The internalized EGFR was confirmed by changed subcellular distribution and co-localization with EEA1, while no effect on the viral entry of internalized EGFR was found in JEV-infected hBMECs. The inconsistent result of EGFR in different virus infections is not surprising, which also appeared in the previous study. During human cytomegalovirus (HCMV) infection, Wang et al. indicated that EGFR is a necessary cellular receptor for viral entry (Wang et al., 2003). In contrast, others hold an inconsistent view that EGFR is not an essential factor for cellular invasion or virus-induced signaling (Soroceanu et al., 2008). These inconsistencies might be due to the distinct cell types or differential infection conditions, and more work is needed to address this topic.

Repressed DENV replication was observed in human monocytes in utilizing the EGFR-specific inhibitor gefitinib (Duran et al., 2017), which demonstrates a positive correlation between activated EGFR and viral infection. Moreover, the consistent result of reduced viral infection was also acquired in hBMECs with the use of EGFR-specific inhibitors AG1478 or gefitinib. Notably, in the early phase of *S. suis*-infected hBMECs, the AG1478 caused dephosphorylation of EGFR, which may provide specific protection for the brain from inflammatory cytokine/chemokine-induced BBB disruption (Yang et al., 2016). Further evidence for a decreased production of virions was provided by the EGFR-KO hBMECs with the boosted response of interferon signaling when compared to the WT hBMECs in JEV infection, which showed that activated EGFR weakens the antiviral defense via restraining interferon signaling. Moreover, and not coincidentally, activated EGFR has also been identified to be involved in the production of interferon signaling molecules in other cases, such as cancer and viral infections (Kalinowski et al., 2014; Liu and Han, 2019). The expression of interferon regulatory factor 1 (IRF1) was downregulated by the activation of EGFR signaling in EGFR-mutated non–small cell lung cancer (Sugiyama and Togashi, 2020). In PEDV infection, the activation of EGFR signaling negatively regulates the antiviral activity of interferon (Yang et al., 2018). Even in COVID-19 treatment, targeting EGFR signaling was considered to be an attractive strategy, as its inhibitors may synergistically potentiate the anti-SARS-CoV-2 activity of IFN-I (Matsuyama et al., 2020). Moreover, the IAV-induced activation of EGFR can suppress the production of IFN-λ (Ueki et al., 2013). The result supports the notion, herein, that JEV induced the activation of EGFR, which negatively regulated the production of interferon and helped the virus escape from the antiviral immunity of host cells.

Currently, the EGFR downstream cascade in JEV-infected hBMECs is still enigmatic. Activated EGFR recruits several major downstream signaling pathways during viral infection. Yang et al. elucidated that the depleted EGFR strengthens the host antiviral activity, which requires attenuated STAT3 signaling (Yang et al., 2018). A more recent model indicated that ZIKV infection induces the activation of EGFR and further transduction of the MAPK/ERK signaling cascade (Sabino et al., 2021). Intriguingly, the phosphorylation of STAT3 and ERK was noticed in both JEV- and rhEGF-treated hBMECs. Therefore, the question arises as to which of the two proteins acts as the downstream effector of EGFR. As a previous study reported, EGF induces the activation of transcription factor TAZ via EGFR and downstream factors STAT3 and ERK (Gao et al., 2021). By comparing the phosphorylation levels of STAT3 and ERK from cells with or without the treatment of EGFR-specific inhibitors, both STAT3 and ERK were identified as the downstream cascade (Xu et al., 2017), which was further evidenced by utilizing EGFR-KO hBMECs in our study. Then, we explored which downstream factor, STAT3 or ERK, is involved in the host's innate immunity in resisting JEV infection. Inhibition of STAT3 by 17-AAG in hBMECs efficiently impaired JEV infection, which was not observed in MAPK/ERK inhibition by U0126, supporting that the MAPK/ERK pathway could contribute to the JEV life cycle, which is similar to the result in ZIKV infection (Sabino et al., 2021). The previous report demonstrated that the MEK1/2-ERK pathway negatively regulates interferon production in plasmacytoid dendritic cells (Janovec et al., 2018). In human microvascular endothelial cells, ERK was considered a contributor to rosiglitazone-inhibited IFN-γ production (Lombardi et al., 2008). Similar results in U0126-treated hBMECs illustrated a noticeable increase in IFNs and ISGs, which hinders immune escape to JEV in this study. Consistently, activation of the ERK pathway impaired the antiviral activity of IFN-α in resisting HCV infection, indicating a complex relationship between ERK and IFNs (Zhao et al., 2015). The treatment of AG1478 or U0126 was also demonstrated to lighten bile acid prompted HCV replication and recover the anti-HCV effects by inducing IFN-α in replicon-harboring cells (REF) (Patton et al., 2011). In IAV infection, activated EGFR/ERK signaling was also evidenced to suppress type I and type III interferon-mediated host antiviral innate immunity (Wang et al., 2021). Thus, the EGFR/ERK signaling pathway hijacked by viruses might be a common immune escape strategy for viral infection.

In conclusion, our result in this study for the first time provides new insight into the mechanism that JEV-induced activation of EGFR-ERK signaling cascade contributes to viral escape from host innate immunity by suppressing the interferon signaling response in hBMECs. JEV infection induced EGFR activation, manifesting in promoting the phosphorylated form of EGFR and further contributing to EGFR internalization. Indeed, blockade of EGFR signaling with EGFR inhibitors reduced JEV replication and impeded viral particle production. Further study demonstrated that the positive correlation between EGFR phosphorylation and viral replication was mainly dependent on ERK phosphorylation. The ERK inhibitor substantially alleviated the constraint of interferons and ISGs and lowered viral replication. Taken together, it is worthwhile to consider EGFR as a potential target for antiviral strategies in the future.

## Data Availability Statement

The original contributions presented in the study are included in the article/Supplementary Material, further inquiries can be directed to the corresponding author.

## Author Contributions

Y-GZ conceived and performed the experiments, analyzed the data, and drafted the manuscript. H-WC, H-XZ, and KW analyzed the data and revised the manuscript. Y-RC and JS assisted to complete the experiment. X-RW and Z-FF provided the material and technological support. MC conceived the experiments and revised the manuscript. All authors contributed to the article and approved the submitted version.

## Funding

The authors are grateful for the financial support provided by the National Natural Sciences Foundation of China (Grant No. 31872455) and the Natural Sciences Foundation of Hubei Province (2019CFA010).

## Conflict of Interest

The authors declare that the research was conducted in the absence of any commercial or financial relationships that could be construed as a potential conflict of interest.

## Publisher's Note

All claims expressed in this article are solely those of the authors and do not necessarily represent those of their affiliated organizations, or those of the publisher, the editors and the reviewers. Any product that may be evaluated in this article, or claim that may be made by its manufacturer, is not guaranteed or endorsed by the publisher.

## Abbreviations

JEV: Japanese encephalitis virus
EGFR: epidermal growth factor receptor
BBB: blood–brain barrier
BMECs: brain microvascular endothelial cells
hBMECs: human brain microvascular endothelial cells
CNS: central nervous system
RNA-Seq: RNA sequencing
qRT-PCR: quantitative reverse transcription-polymerase chain reaction
PPIs: protein–protein interactions
DMEM: Dulbecco's Modified Eagle's Medium
FBS: fetal bovine serum
PFU: plaque-forming units
IFN: interferon
MOI: multiplicity of infection
IF: immunofluorescence
WB: Western blotting
hpi: hours post-infection
ISGs: interferon-stimulated genes.
