# Supplementary material for: EGFR Activation Impairs Antiviral Activity of Interferon Signaling in Brain Microvascular Endothelial Cells During Japanese Encephalitis Virus Infection
Source: Front Microbiol. 2022 Jun 30;13:894356. doi: 10.3389/fmicb.2022.894356 (PMC9279666; doi:10.3389/fmicb.2022.894356)
Supplement: Supplementary file 1 [file Data_Sheet_1.PDF]

Supplementary Material

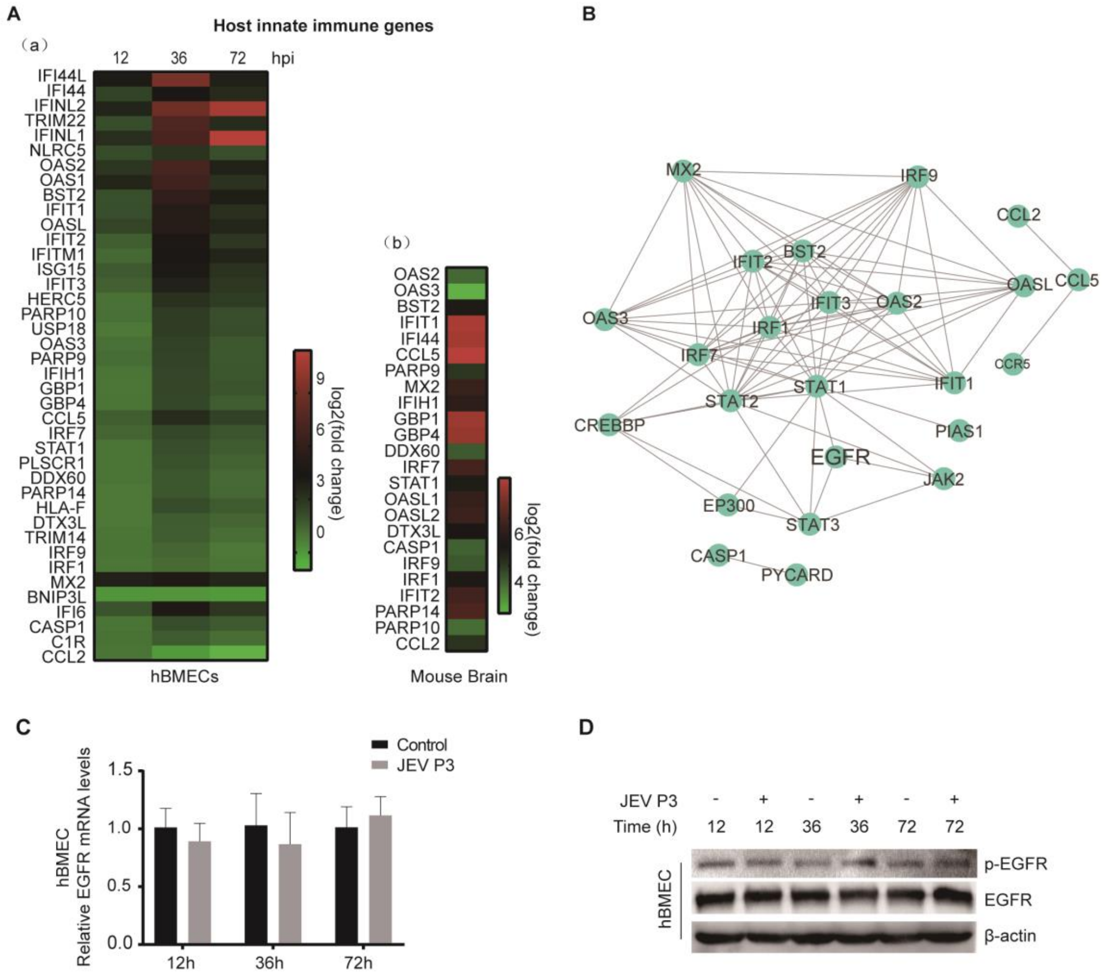

Supplementary Figure 1. EGFR was determined to participate in host innate pathways based on RNA sequencing results. (A-a) The heat map represents the dynamic regulation of host innate immune genes based on GO analysis of RNA-seq data in JEV P3 infected hBMECs. (A-b) The heat map showed host innate immune genes upregulated in both hBMEC and mouse brain in JEV P3 infection. (B) Cytoscape software and STRING 11.0 were utilized to predict protein to protein interaction (PPI) relationships of selected host innate immune-related genes. (C) hBMECs were infected with JEV P3 or mock-infected for corresponding times, and the cell lysis was harvested.

Real time PCR assay was performed to measure the mRNA expression of EGFR. The protein expression of p-EGFR and EGFR were determined by western blotting analysis with specific antibodies. The β-actin was detected as a loading control. The results are one representative data of three independent experiments and shown as means ± SEM.

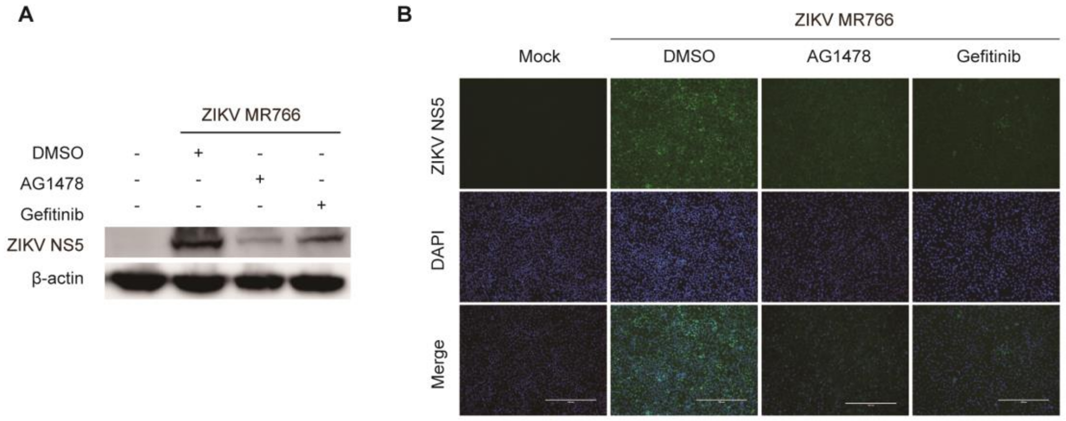

Supplementary Figure 2. EGFR inhibitors reduce Zika virus (ZIKV) infection in hBMECs. hBMECs were pretreated with the carrier control DMSO, AG1478, or Gefitinib for 2 h followed by virus infection for another 24 h. (A) The western blotting assay was performed to measure the ZIKV viral protein expression with ZIKV non-structural protein 5 (NS5) monoclonal antibody. (B) The immunofluorescence (IF) assay was performed to determine ZIKV infection in hBMECs at 24 hpi with the specific antibody of ZIKV NS5. Nuclei were stained with DAPI, Scale bars, 400 μm. The results are the representative data of three independent experiments.

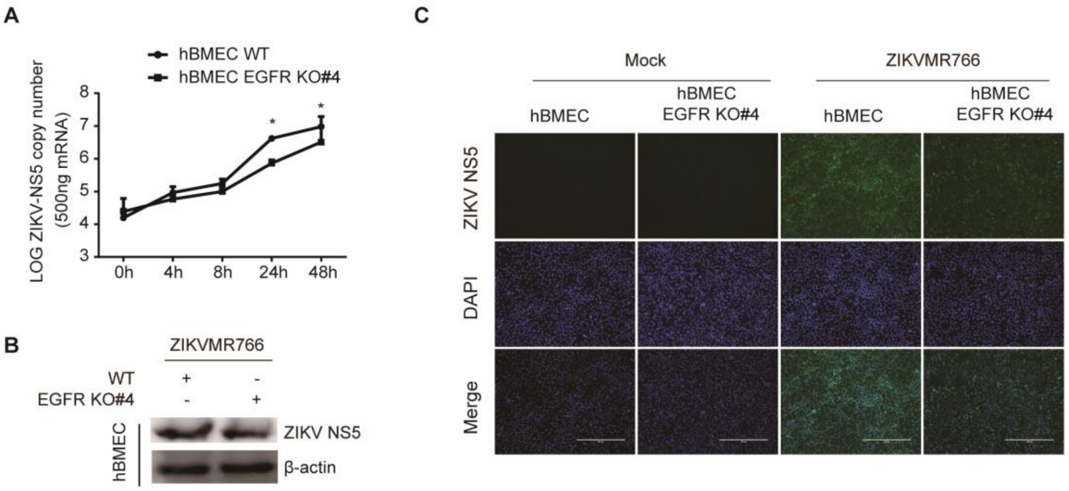

Supplementary Figure 3. The knockout of endogenous EGFR attenuates ZIKV infection in hBMECs. (A) The comparison of ZIKV infection in RNA levels between hBMECs and EGFR KO hBMECs over time. (B) Western blotting assay was performed to determine ZIKV infection in wild-type and EGFR KO hBMECs with the specific antibody of ZIKV NS5. (C) Wild-type and EGFR KO hBMECs were infected with ZIKV for 24h, and then immunofluorescence (IF) staining analysis was performed to detect ZIKV NS5 expression. Nuclei were stained with DAPI. Scale bars, 400 μm. The results are the representative data of three independent experiments.*, $p < 0.05$.
